# A Model to Investigate the Impact of Farm Practice on Antimicrobial Resistance in UK Dairy Farms

**DOI:** 10.1007/s11538-021-00865-9

**Published:** 2021-03-01

**Authors:** Christopher W. Lanyon, John R. King, Dov J. Stekel, Rachel L. Gomes

**Affiliations:** 1 School of Mathematical Sciences, University of Nottingham, Nottingham, NG7 2QL UK; 2 School of Biosciences, University of Nottingham, Loughborough, LE12 5RD UK; 3 Food, Water, Waste Research Group, Faculty of Engineering, University of Nottingham, Nottingham, NG7 2RD UK

**Keywords:** Antimicrobial resistance, Ordinary differential equations, Agriculture, Modelling, Slurry, Antibiotics

## Abstract

The ecological and human health impact of antibiotic use and the related antimicrobial resistance (AMR) in animal husbandry is poorly understood. In many countries, there has been considerable pressure to reduce overall antibiotic use in agriculture or to cease or minimise use of human critical antibiotics. However, a more nuanced approach would consider the differential impact of use of different antibiotic classes; for example, it is not known whether reduced use of bacteriostatic or bacteriolytic classes of antibiotics would be of greater value. We have developed an ordinary differential equation model to investigate the effects of farm practice on the spread and persistence of AMR in the dairy slurry tank environment. We model the chemical fate of bacteriolytic and bacteriostatic antibiotics within the slurry and their effect on a population of bacteria, which are capable of resistance to both types of antibiotic. Through our analysis, we find that changing the rate at which a slurry tank is emptied may delay the proliferation of multidrug-resistant bacteria by up to five years depending on conditions. This finding has implications for farming practice and the policies that influence waste management practices. We also find that, within our model, the development of multidrug resistance is particularly sensitive to the use of bacteriolytic antibiotics, rather than bacteriostatic antibiotics, and this may be cause for controlling the usage of bacteriolytic antibiotics in agriculture.

## Introduction

Antimicrobial resistance (AMR) has long been recognised as a threat to environmental, animal and human health. Recent analysis predicts that by 2050 our inability to treat previously curable diseases will lead to more deaths than cancer (O’Neill 2015). Beyond healthcare, human use of antibiotics means that AMR is now highly prevalent in agriculture and the environment. Though AMR persists at low levels in nature, it is particularly prevalent in circumstances where large quantities of antimicrobials or antibiotics are present, for example in antibiotic manufacturing effluent (Lateef 2004; Cardoso et al. 2014) municipal sewage (Li et al. 2009, 2010) and agricultural runoff (Kümmerer 2004), all of which are sources of AMR to the environment (Berendonk et al. 2015; Thanner et al. 2016).

The human health impact of antimicrobial use in agriculture is poorly understood (Thanner et al. 2016), despite agricultural usage accounting for around 44% of antibiotics by weight in the UK and an estimated 70% in the USA (Department of Health 2014; O’Neill 2015). It has also been shown that the use of antimicrobial agents in agriculture affects the human resistome (Witte 1998; Smith et al. 2002). Furthermore, there is evidence that animal husbandry can directly impact the commensal flora of farmers (Aubry-Damon et al. 2004). In the case of colistin, a so-called last-line or last-resort antibiotic (Nation and Li 2009; Chaudhary 2016), there is evidence that the resistance gene mcr-1 has been transferred to human populations as a result of agricultural use (Liu et al. 2016). This prompts the need for more in-depth research into the effects of farm practice and antibiotic usage on the development of AMR in the farming and recipient environments.

In this paper, we focus on the dairy farm slurry tank, a repository for dairy farm wastewater including bovine faeces and urine, parlour washings, waste milk and waste footbath contents. Antimicrobial resistance genes (ARGs), antimicrobial resistant microbes (ARBs) and antibiotics are discharged by cows (Zhou et al. 2013; Chambers et al. 2015); combining this with the natural presence of bacteria means that the slurry tank is potentially a site for the development and proliferation of AMR. Slurry from the tank is spread onto soils used to grow human and livestock crops.

Cows are treated by vets for illnesses such as mastitis with antibiotics which are often not fully metabolised by the body and are then passed in urine and faeces either unaltered or as active metabolites (Hillerton and Berry 2003; Massé et al. 2014). For example the antibiotic oxytetracycline, used to treat mastitis (MacDiarmid 1978; Erskine et al. 1994; Pyörälä 2009), has been found in concentrations between 0.5 and 200 mg l$^{-1}$ in cow manure (Massé et al. 2014). Dairy manure has also been found to host ARGs related to tetracycline, beta-lactam, kanamycin and chloramphenicol resistance (Wichmann et al. 2014). Contaminated urine, faeces and any milk produced during the treatment period are added to the slurry tank. Additionally, footbaths, once used, are added to the tank (Williams et al. 2019) along with any run-off from parlour washings (AHDB Dairy 2019). These footbaths may contain heavy metals such as copper and zinc and bacteriocides such as formalin (Cornelisse et al. 1982). In nitrate-vulnerable zones (NVZ) slurry (and other organic manures) can be used as a fertiliser and spread on soils used to grow both human and livestock food crops between 1st February and 31st October each year, potentially creating a transmission vector for AMR into the human population (Singer et al. 2016). NVZs make up around 55% of land in the UK (European Community 2001; Department for Environment, Food and Rural Affairs 2018; Department of Agriculture, Environment and Rural Affairs 2019; Department of Health 2017). Agricultural land not deemed to be in an NVZ may have more lenient spreading restrictions.

As farm slurry spread on agricultural land may be a route for pathogens into the human food chain (Nicholson et al. 2005), the extent to which the slurry tank environment drives the development and spread of AMR is of particular interest from a human health perspective. A greater understanding of on-farm dynamics is also important for effecting policy change to better protect against the risks of AMR developing within agriculture. Though there is government policy in place intended to combat the development of agricultural AMR, it is mostly concerned with the reduction of antibiotic use, which is a high priority (Post Report 588 2018). For example, in the UK (and EU), antibiotics cannot be used as growth promoters (Castanon Jan 2007; Maron et al. 2013; Hao et al. 2014). Furthermore, in 2018 the EU approved legislation banning the use of antibiotics as prophylactics in farming, to come into force in 2022 (Farming Monthly 2018). The existing UK policy around slurry storage and spreading is generally intended to manage soil nutrient levels (Department of Agriculture, Environment and Rural Affairs 2019) and not AMR, despite the fact that there is evidence that run-off from farms and manure-fertilised fields may result in the contamination of the local environment (Swift et al. 2019). The Food Standards Agency (FSA) provides guidance for slurry storage and management with the aim of reducing pathogen transfer to ready to eat foods, though there is no mention of AMR and farmers are not obligated to follow the guidance (Food Standards Agency 2009). Though the FSA advises storing slurry for longer to reduce the number of pathogens in the tank, it is not known what the effects of long storage times are on the prevalence and spread of AMR. As policy influences farm practice, if there are slurry storage processes, such as tank filling and emptying, which are increasing the risk of agricultural AMR, it is important that they are understood so that policy can be put into place appropriately.

In the UK, the Responsible Use of Medicines in Agriculture Alliance (RUMA) set a series of targets for reducing the use of antibiotics in eight sectors of agriculture in 2017, including the dairy sector (RUMA 2017). These targets aim to both reduce the total quantity of antibiotics used, and limit the use of antibiotics that are of a high priority for human health. By the end of 2018, the dairy sector reported a 35% reduction in total antibiotics used, bringing it under the proposed target of a 20% reduction by 2020. RUMA refer to this as “significant progress” (RUMA 2018) and also note that both the beef and dairy networks formed stewardship groups that are working together to efficiently manage antibiotic usage in cattle. Other sectors, such as pig farming, did not meet their overall targets, but did limit the use of high-priority antibiotics (RUMA 2018). The recommended reductions are across the board. However, it may be that reducing the use of certain classes of antibiotic has more effect on controlling the development of AMR.

The antibiotics used in agriculture can be broadly defined as either bacteriolytic (those that kill bacteria) and bacteriostatic (those that inhibit the reproduction of bacteria); it is possible that reducing one of these types may be an effective way of controlling AMR. This possibility has never been investigated before, and it is a key part of this work to try and predict the differential impact of such potential reductions.

Mathematical modelling can be used to quickly and efficiently identify important process factors using computer simulation. This is of particular relevance in agriculture, since altering farming practice on working farms is often untenable. Particularly in the case of slurry storage, where practical experiments may conflict with a farmer’s needs, models can be used to simulate changes in farm practice over a number of years without having to enact those changes. Furthermore, there is a lot of variability from farm to farm, which makes easily adaptable models especially useful.

Existing mathematical models of AMR in agriculture often model the emergence of a single resistant pathogen (or treat all pathogens as a homogenous group). The single pathogen is often considered only in the context of a single antibiotic and does not take into account the use of other antimicrobials or the wider environmental context which is widely ignored, despite the fact that variables such as temperature and seasonality affect the development of AMR (Dolliver et al. 2008; Sui et al. 2015). Existing agricultural models can be split into two categories: those that model the effect of antibiotic use in agriculture on humans and those that model the spread and prevalence of AMR in agricultural systems. Epidemiological models have also been employed to modelling the spread of agricultural AMR bacteria between humans (Smith et al. 2002; Van Bunnik and Woolhouse 2017). These conclude that the quantity of antibiotics used in agriculture has little effect on the prevalence of AMR in humans. Smith et al. (2002) also posit that agricultural antibiotic usage may hasten the development of AMR in humans, despite having little effect on the prevalence of AMR in the human population (Smith et al. 2002), while the reduction of human-animal AMR transmission has been posited as a potential method for reducing AMR in humans (Van Bunnik and Woolhouse 2017). The work of Volkova et al. (2012) and Baker et al. (2016) models the spread and prevalence of AMR in cow stomachs and dairy slurry tanks, respectively. In these models the variable of interest is the level of resistant bacteria within the system; both conclude that one of the biggest factors for AMR prevalence is the rate of transfer of resistance genes between microbes.

Dairy slurry tanks are replete with many different bacterial strains and various different antibiotics. These antibiotics are generally either bacteriolytic or bacteriostatic and bacteria can acquire separate resistance methods for different antibiotics via resistance plasmids (Bennett 2008). Multidrug resistance (MDR) is known to be common in dairy slurry (Ibrahim et al. 2016). Little is currently known about whether either type of antibiotic contributes more to the development of MDR. This prompts the development of a mathematical model intended to predict the evolution of *E. coli* within a slurry tank that are capable of both bacteriolytic and bacteriostatic resistance and can be resistant to one or the other type of antibiotic, or resistant to both.

## Materials and Methods

The ordinary differential equation (ODE) model detailed here was designed to examine the conditions under which MDR bacteria are present, absent or dominant in the slurry tank environment and, through sensitivity analysis, to identify process factors that contribute to the development of AMR and the relative importance of different classes of antibiotics to the prevalence and spread of MDR.

The model draws on those developed by Volkova et al. (2012) and Baker et al. (2016) but differs significantly in that it encompasses twice as many antibiotics and bacterial strains, incorporates a mechanism for antibiotic-induced cell lysis, and explicitly incorporates a tank filling and emptying regime, thus providing a more detailed model of the dairy slurry tank system.

### Model Assumptions

Our assumptions when designing this model aim to mimic the real-world scenario while providing an appropriate level of simplification. We base our model on a farm with a herd of approximately 200 dairy cows, with each cow producing approximately 63kg of waste per day (AHDB Dairy 2019).

We assume that the slurry in the tank is well mixed by a rotor mixer and operating as a completely stirred tank reactor, meaning that bacteria, nutrients and antibiotics are evenly distributed throughout the tank, as described by Baker et al. (2016). This assumption eliminates any spatial element from the model, simplifying the equations required to describe the dynamics. We also assume that the tank in the model is uncovered (AHDB Dairy 2019) but that dilution by rainfall is negligible given the volume of the reactor, so that the entirety of inflow to the tank is slurry from the farm combined with other farm wastewater (parlour washings, waste footbath contents and waste milk (AHDB Dairy 2019)) and that the inflow of slurry occurs at a constant rate. For simplicity, the quantity of antibiotics, bacteria and the relative proportions of resistant and susceptible bacteria in the inflow also remain constant. Antibiotic inflow is assumed to occur at a constant rate due to regular veterinary prescriptions, as in Baker et al. (2016). This is assumed true for both bacteriostatic and bacteriolytic antibiotics.

We assume that both bacteriostatic and bacteriolytic antibiotics are deposited into the tank (Economou and Gousia 2015). By combining certain veterinary antibiotics their mode of action can be changed. For example, though trimethoprim is bacteriostatic, when combined with sulphonamides it becomes bacteriolytic (Masters 2016). However, for simplicity, we assume that there is no interaction between the two antibiotics. For both antibiotics we assume that the rate of change of the concentration is determined by three factors, inflow of antibiotic into the slurry tank, antibiotic degradation and the changing volume of the tank.

We assume that the rate of change of concentration of each strain of *E. coli* depends on population growth, following a logistic growth model (Edelstein-Keshet 2005); acquisition of resistance genes via HGT (Davies and Davies 2010); natural cell death (Ayscue et al. 2009); and antibiotic induced cell death (Spalding et al. 2018).

### Model Design and Parameterisation

#### The Slurry Tank

The volume of slurry within the tank (*V*(*t*)) is modelled with the equation

1 $$\begin{aligned} V(t) = V_1+\varLambda (t) -\omega (t). \end{aligned}$$

where $V_1$ is the initial volume of slurry in the tank, $\varLambda (t)$ is the quantity of slurry flowing into the tank and $\omega (t)$ is the quantity of slurry flowing out of the tank. For our initial simulations we assume that the tank fills at a constant rate, has infinite capacity and is never emptied, as a simple first case and following the Baker model Baker et al. (2016). This gives $\varLambda (t)$=$\lambda t$ where $\lambda$ is the rate of inflow and $\omega (t)=0$. We set $\lambda = 613\hbox {lh}^{-1}$, from a 200 cow herd. When slurry tanks are emptied, there is often a small amount of slurry left in the tank, for consistency with Baker et al. (2016) we assume that the minimum quantity of slurry in the tank is 150,000 l, so $V(t) = 150,000 + \lambda t$. Later in our analysis alternative filling and emptying regimes are considered.

As slurry flows into the tank, the concentration of antibiotics and bacteria changes, along with the volume of slurry within the tank. This is accounted for in the model by a so-called volume change term:

2 $$\begin{aligned} -C(t)\frac{\varLambda '(t)}{V(t)}, \end{aligned}$$

where *C*(*t*) is the concentration of some substance *Q*(*t*). This term can be derived from an ODE for the quantity of *Q*(*t*) in the tank, where the change in *Q*(*t*) is determined by some reaction term *G*(*t*), the influent rate of *Q*(*t*), *I*, and the effluent rate of *Q*(*t*), given by $\omega '(t)Q(t)/V(t)$. The ODE for *Q*(*t*) is then given by

3 $$\begin{aligned} \frac{\mathrm{d}Q}{\mathrm{d}t} = G(t)Q(t) + I - \omega '(t)\frac{Q(t)}{V(t)}. \end{aligned}$$

This is analogous to the ODE models used by Baker et al. (2016) and Volkova et al. (2012). As *C*(*t*) is the concentration of *Q*(*t*), $C(t)=Q(t)/V(t)$ and

4 $$\begin{aligned} \frac{\mathrm{d}C}{\mathrm{d}t} = \frac{\mathrm{d}}{\mathrm{d}t}\left( \frac{Q(t)}{V(t)}\right) = \frac{Q'(t)V(t) - Q(t)V'(t)}{V^2(t)} = \frac{Q'(t)}{V(t)} - C(t)\frac{V'(t)}{V(t)}. \end{aligned}$$

Then, substituting in the known value of $Q'(t)$ from Eq. 3 and the derivative of Eq. 1,

5 $$\begin{aligned} \frac{\mathrm{d}C}{\mathrm{d}t}&= \frac{G(t)Q(t) + I}{V(t)} - C(t)\frac{\omega '(t)}{V(t)}- C(t)\frac{\varLambda '(t) -\omega '(t)}{V(t)} = G(t)C(t) + \frac{I}{V(t)}\nonumber \\&\quad - C(t)\frac{\varLambda '(t)}{V(t)}. \end{aligned}$$

The last term of Eq. 5 is the volume change term.

#### Antibiotics

Though the modes of action of bacteriostatic and bacteriolytic antibiotics vary within their respective classes, the resultant effects of each class remain the same. In the case of bacteriostatic antibiotics, reproduction is inhibited, slowing population growth. This is modelled by modifying a logistic growth term using a Hill function, following Volkova et al. (2012) and Baker et al. (2016). Bacteriolytic antibiotics on the other hand lead to cell death, which is modelled by incorporating a death term into the equations, based on Michaelis–Menten kinetics (Spalding et al. 2018). We chose to include both dynamics into our model, in order to analyse whether reproduction inhibition or cell death had a greater effect on the development and survival of resistant bacteria.

We assume a total antibiotic inflow rate of $3422\mu \hbox {gh}^{-1}$ following Baker et al. (2016). For simplicity we assume that the total rate of antibiotic inflow is split evenly between bacteriolytic and bacteriostatic antibiotics so that $\theta _s = \theta _l = 1711\mu \hbox {gh}^{-1}$. We assume that both bacteriostatic and bacteriolytic antibiotics undergo first order degradation at constant rates ($\gamma _s, \ \gamma _l$, respectively) as described by Volkova et al. (2012); Baker et al. (2016) and Spalding et al. (2018). Van Epps and Blaney (2016) found that the half-life of the bacteriostatic antibiotic Oxytetracycline varied between 3 and 31 days in beef and dairy cattle manure compost, specifically 9.8 and 17.7 days in the two dairy manure samples. The antibiotic trimethoprim, which is bacteriostatic on its own but becomes bacteriolytic in the presence of sulphonamides, with which it is often combined, has a half-life in manure-amended soils varying between 2.3 and 197 days, depending on soil type and whether the system was anaerobic or aerobic, in general the half-life was shorter in the anaerobic case (Wu et al. 2012). Though the half-life of cephalosporins (a widely used bacteriolytic antibiotic) has not been recorded in dairy slurry, Jiang et al. (2010) found that cephalosporins have half-lives varying between 2.7 and 18.7 days in lake surface water. For simplicity we assume that both antibiotics decay at the same rate and have a half-life of 10 days, equating to a rate of decay of $\gamma _s = \gamma _l = 0.0029\,\,\hbox {h}^{-1}$. These parameters were allowed to vary independently during sensitivity analysis.

#### Bacteria

For our model we chose *Escherichia coli* (*E. coli*) as a model organism. *E. coli* is found in dairy cow manure (Sawant et al. 2007), is a common cause of mastitis in dairy cows (AHDB Dairy 2020) and is often regarded as a sentinel organism for monitoring AMR (Tadesse et al. 2012). However, there are likely high levels of microbial diversity in the slurry tank and this model can easily be adjusted to simulate the growth of bacteria.

The growth of bacterial communities is classically modelled using logistic growth (Edelstein-Keshet 2005). For our model, following Volkova et al. (2012); Baker et al. (2016), we model microbial growth using a logistic growth term modified by a Hill function, which accounts for the effects of bacteriostatic antibiotics. Cutler (2016) found that the growth-rate of *E. coli* in manure-amended soils varied between 0.05 and 0.6 $\hbox {h}^{-1}$; Kim et al. (2009) found that rifampin-resistant *E. coli* had a growth rate of 0.33 $\hbox {h}^{-1}$ in a 2:1 compost and water growth media; and Baker et al. (2016) note that faster growth rates have been recorded in laboratory conditions (up to 0.9 $\hbox {h}^{-1}$) by Godwin and Slater (1979) and Levin et al. (1979), and a value of 0.5 $\hbox {h}^{-1}$ has previously been used to model the growth of *E. coli* in activated sludge (Curds 1971). Using this information and following Baker et al. (2016) we chose a value of $r=0.5$ $\hbox {h}^{-1}$ for the specific growth rate.

In the model, bacteria can become resistant to either or both antibiotics, allowing for the emergence of MDR (Davies and Davies 2010). Rather than considering stochastic transfer events, we homogenise these into a constant rate of horizontal gene transfer (HGT), after Baker et al. (2016). We assume that the strains of bacteria can acquire new genetic elements which confer resistance through HGT (Davies and Davies 2010). We have simplified the plasmid and gene dynamics here, assuming that the relevant genes for resistance to both antibiotics have become associated on a single mobile genetic element (in the case of susceptible bacteria becoming multidrug resistant with no intermediate resistances being acquired). We have also assumed that plasmids carrying individual resistance genes are compatible (Novick 1987).

HGT is described in the model by adapting an approach from chemical reaction kinetics. For a reaction of the type

6 $$\begin{aligned} A + B \xrightarrow {k} B + C, \end{aligned}$$

where *A* and *B* are chemical reactants and *C* is the reaction product, the reaction kinetics for the *C* can be described mathematically by employing the *law of mass action*, which results in the differential equation

7 $$\begin{aligned} \frac{\mathrm{d}[C]}{\mathrm{d}t} = k[A][B] \end{aligned}$$

where square brackets indicates the concentration of each chemical (Murray 2002). Under the assumption that the conversion of susceptible bacteria to resistant bacteria is irreversible, the interaction between the two microbial strains can be considered as a reaction equation:

8 $$\begin{aligned} S_t + R_t \xrightarrow {k_\beta } 2R_t, \end{aligned}$$

where $S_t$ and $R_t$ are the quantities in colony forming units (CFU) of susceptible and resistant strains of bacteria, respectively, and $k_\beta$ is the rate of HGT. Positing that this applies to all three strains of resistant bacteria, using (7) implies a gene transfer term:

9 $$\begin{aligned} S(\beta _sR_s +\beta _lR_l + \beta _uR_u) \end{aligned}$$

where *S* is the concentration of susceptible bacteria; $R_s$, $R_l$ and $R_u$ are the concentrations of bacteriostatic, bacteriolytic and multidrug-resistant bacteria, respectively; and $\beta _s$, $\beta _l$ and $\beta _u$ are the HGT rates for bacteriostatic, bacteriolytic and multidrug-resistant bacteria, respectively, each with dimensions of l $\hbox {CFU}^{-1}$ $\hbox {h}^{-1}$. The three rates of HGT, $\beta _s$, $\beta _l$ and $\beta _u$ are all initially set to $10^{-14}$, based on the mass-action rates of transfer given in Zhong et al. (2010), which are equivalent to between $10^{-12}$ and $10^{-16}$ $\mathrm {l CFU}^{-1}\mathrm {h}^{-1}$. In Volkova et al. (2012); Baker et al. (2016), the HGT process is modelled using a “force of transfer” term: $\beta RN^{-1}$, where $\beta$ is the rate of transfer, *R* is the number of resistant bacteria, and N is the total number of bacteria. As in Baker et al. (2016) the bacterial population quickly grows to reach carrying capacity, $\beta RN^{-1} \sim \beta R\mu ^{-1}$. Our mass-action rate of transfer leads to the equivalent of an order of magnitude smaller than the rates of HGT used by Volkova et al. (2012) and Baker et al. (2016) (0.001 and 0.004, respectively) but well within the range of possible values described in Baker et al. (2016). During sensitivity testing $\beta _u$, $\beta _l$ and $\beta _s$ were allowed to vary to investigate the impact of faster and slower genetic transfer.

Minimum inhibitory concentrations (MIC) vary greatly between antibiotics. For our model we chose the MIC of bacteriostatic antibiotics, (MIC$_s$), to be $8\ \mu \mathrm {g \ l}^{-1}$, based on the size-adjusted MIC for trimethoprim, a bacteriostatic antibiotic used in agriculture (Economou and Gousia 2015), reported by Bengtsson-Palme and Larsson (2016) and slightly higher than the adjusted MIC for oxytetracycline, another commonly used veterinary antibiotic. The MIC of bacteriolytic antibiotics (MIC$_l$) is set to $4\ \mu \mathrm {g \ l}^{-1}$, the size-adjusted MIC of bacteriolytic veterinary antibiotic ampicillin (Bengtsson-Palme and Larsson 2016; Economou and Gousia 2015). This MIC is also a similar order of magnitude to several cephalosporin antibiotics, bacteriolytic antibiotics which frequently used in agriculture (Bengtsson-Palme and Larsson 2016; Economou and Gousia 2015).

We assume that bacteria incur a relative fitness cost for resistance to each type of antibiotic (Melnyk et al. 2015). We assume that these costs, $\alpha _s$, $\alpha _l$ and $\alpha _u$, are all equal, i.e. no extra cost is incurred by multidrug-resistant bacteria, due either to selective pressure reducing the cost of MDR, or the presence of a compensatory mutation. Furthermore, the resistance fitness costs are all constant and within the range for *E. coli* described in Melnyk et al. (2015) at $\alpha _s=\alpha _l=\alpha _u=0.1$.

We assume that bacteria can die from both natural causes and from exposure to bacteriolytic antibiotics (Norcia et al. 1999). Following Ayscue et al. (2009), we assume that natural death occurs at a constant rate and the natural death rate for susceptible and resistant bacteria are denoted by $\delta _{S/R}$, the proportional death rate per hour. Following Jiang et al. (2002); McGee et al. (2001); Maule (2000) and (Ayscue et al. 2009), the natural death rates for susceptible and resistant bacteria, $\delta _{S}$ and $\delta _{R}$, are set to 0.001$\hbox {h}^{-1}$. For bacteria susceptible to bacteriolytic antibiotic, following Spalding et al. (2018), the antibiotic induced death rate is described using Michaelis–Menten kinetics:

10 $$\begin{aligned} \varDelta _A = \delta _l \frac{A_l}{\mathrm{MIC}_l + A_l}, \end{aligned}$$

where $\delta _l$ is the rate at which antibiotic kills bacteria at MIC, $A_l$ is the concentration of bacteriolytic antibiotic and MIC$_l$ is the minimum inhibitory concentration of the bacteriolytic antibiotic. Initially the antibiotic induced death rate, $\delta _l$, was set to 0.01 (at the same order of magnitude as Spalding et al. (2018)’s initial parameter estimate of 0.06 $\hbox {h}^{-1}$), corresponding to a low death rate due to bacteriolytic antibiotic. However, as enrofloxacin and penicillin are both used in dairy agriculture (Economou and Gousia 2015) and have $\delta _l\approx 3$ at eight times MIC in vitro (Norcia et al. 1999), $\delta _l$ was subsequently varied between 0 and 3 during sensitivity analysis.

Baker et al. (2016) report *E. coli* concentrations between 2 and 6$\times 10^4$ CFU $\hbox {ml}^{-1}$ in dairy slurry, consistently within the same range reported by Reinthaler et al. (2003). Furthermore, the proportion of drug-resistant strains of *E. coli* in healthy lactating dairy cows is estimated to be approximately 40% (Sawant et al. 2007). Using these values, assuming there are no MDR bacteria present in the cows and once again following Baker et al. (2016), we choose the concentration of bacteria in the inflow to be $\nu =6\times 10^7\hbox {CFUl}^{-1}$ and and the proportions of bacteriostatic, bacteriolytic and multidrug-resistant bacteria in the inflow, respectively, $\rho _s=\rho _l=0.2$ and $\rho _u=0$, so that the total proportion of resistant bacteria in the inflow is 0.4.

Though temperature has been shown to affect the survival of pathogens in slurry, Kearney et al. (1993) recorded no difference in *E. coli* population decimation time between samples stored in 4$^{\circ }$C and 17$^{\circ }$C cattle slurry. Similarly Biswas et al. (2016) showed that *E. coli* populations in slurry (which ranged between 30$^{\circ }$C and 50$^{\circ }$C) had increased survivability at varying temperatures compared to *Salmonella spp.* and *Listeria monocyogenes*, with *E. coli* populations only declining slightly over the incubation period. Due to this we omit temperature dependence from our model, though it may be important to have a temperature dependent death rate when modelling other bacteria.

All parameter definitions and typical values are shown in Table 1.

**Table 1:** Typical parameter values used for initial simulations of the model

Parameter	Description	Typical value	References
*r*	Specific growth rate of *E. coli*	$0.5\ \mathrm {h}^{-1}$	Baker et al. (2016)
$\beta _s$	Bacteriostatic-resistant gene transfer term	$10^{-14}\ \mathrm {l CFU}^{-1}\mathrm {h}^{-1}$	Zhong et al. (2010), Volkova et al. (2012), Baker et al. (2016)
$\beta _l$	Bacteriolytic-resistant gene transfer term	$10^{-14}\ \mathrm {l CFU}^{-1}\mathrm {h}^{-1}$	Zhong et al. (2010), Volkova et al. (2012), Baker et al. (2016)
$\beta _u$	MDR gene transfer term	$10^{-14}\ \mathrm {l CFU}^{-1}\mathrm {h}^{-1}$	Zhong et al. (2010), Volkova et al. (2012), Baker et al. (2016)
$\lambda$	Rate of slurry inflow to tank	613$\mathrm {l h}^{-1}$	Baker et al. (2016)
$\nu$	Concentration of bacteria in slurry inflow	$6\times 10^7\ \mathrm {CFU \ l}^{-1}$	Reinthaler et al. (2003), Baker et al. (2016)
$\rho _s$	Proportion of bacteriostatic-resistant bacteria in inflow	0.2	Sawant et al. (2007)
$\rho _l$	Proportion of bacteriolytic-resistant bacteria in inflow	0.2	Sawant et al. (2007)
$\rho _u$	Proportion of multidrug-resistant bacteria in inflow	0.0	Sawant et al. (2007)
$\alpha _s$	Bacteriostatic-resistant fitness cost as a fraction of *r*	0.1	Melnyk et al. (2015), Baker et al. (2016)
$\alpha _l$	Bacteriolytic-resistant fitness cost as a fraction of *r*	0.1	Melnyk et al. (2015), Baker et al. (2016)
$\alpha _u$	MDR fitness cost as a fraction of *r*	0.1	Melnyk et al. (2015), Baker et al. (2016)
$V_1$	Initial volume of slurry in the tank	150,000 l	Baker et al. (2016)
$\theta _s$	Rate of bacteriostatic antibiotic inflow	$1711\ \mu \mathrm {g \ h}^{-1}$	Baker et al. (2016)
$\theta _l$	Rate of bacteriolytic antibiotic inflow	$1711\ \mu \mathrm {g \ h}^{-1}$	Baker et al. (2016)
$E_{\max }$	Maximum effect of antibiotics on bacterial growth	2	Volkova et al. (2012), Baker et al. (2016)
*H*	Hill coefficient in $E_{\max }$ model	2	Volkova et al. (2012); Baker et al. (2016)
MIC$_R$	MIC for bacteriostatic-resistant bacteria	$2000\ \mu \mathrm {g \ l}^{-1}$	Baker et al. (2016)
MIC$_s$	MIC for bacteriostatic-sensitive bacteria	$8\ \mu \mathrm {g \ l}^{-1}$	Bengtsson-Palme and Larsson (2016)
MIC$_l$	MIC for bacteriolytic-sensitive bacteria	$4\ \mu \mathrm {g \ l}^{-1}$	Bengtsson-Palme and Larsson (2016)
$\mu$	Carrying capacity of liquid slurry	$10^{10}\ \mathrm {CFU \ l}^{-1}$	Baker et al. (2016)
$\gamma _s$	Decay rate of bacteriostatic antibiotics	$0.0029\ \mathrm {h}^{-1}$	Baker et al. (2016)
$\gamma _l$	Decay rate of bacteriolytic antibiotics	$0.0029\ \mathrm {h}^{-1}$	Baker et al. (2016)
$\delta _S$	Natural death rate of susceptible bacteria	$0.001\ \mathrm {h}^{-1}$	Ayscue et al. (2009)
$\delta _R$	Natural death rate of resistant bacteria	$0.001\ \mathrm {h}^{-1}$	Ayscue et al. (2009)
$\delta _l$	Antibiotic-driven death rate of bacteriolytic-susceptible bacteria	$0.01\ \mathrm {h}^{-1}$	Norcia et al. (1999), Spalding et al. (2018)

### Mathematical Model

The model is made up of six coupled differential equations, two of which describe the concentrations of bacteriostatic and bacteriolytic antibiotics ($A_s$ and $A_l$), respectively, and four that describe susceptible (*S*), bacteriostatic-resistant ($R_s$), bacteriolytic-resistant ($R_l$) and multidrug-resistant ($R_u$) bacteria.

#### Model Equations

The equations for the antibiotics are as follows:

11 $$\begin{aligned} \frac{\mathrm{d}A_s}{\mathrm{d}t} = \underbrace{\frac{\theta _s}{V(t)}}_\text {Inflow}-A_s\left( \underbrace{\gamma _s}_\text {Degradation}+\underbrace{\frac{\varLambda '(t)}{V(t)}}_\text {Volume change}\right) \end{aligned}$$

and, similarly,

12 $$\begin{aligned} \frac{\mathrm{d}A_l}{\mathrm{d}t} = \frac{\theta _l}{V(t)}-A_l\left( \gamma _l+\frac{\varLambda '(t)}{V(t)}\right) . \end{aligned}$$

The ODEs for the four strains of bacteria are

13 $$\begin{aligned}&\begin{aligned} \frac{\mathrm{d}S}{\mathrm{d}t}&= \underbrace{r\left( 1-\frac{N}{\mu }\right) E_SS}_\text {Modified logistic growth} - \overbrace{S(\beta _sR_s + \beta _lR_l + \beta _uR_u)}^\text {Gene transfer} + \underbrace{\varLambda '(t)(1-\rho )\frac{\nu }{V(t)}}_\text {Inflow} - \overbrace{\frac{\varLambda '(t) S}{V(t)}}^\text {Volume } - \\&\underbrace{\delta _SS}_\text {Natural death}-\overbrace{\delta _lS\frac{A_l}{\mathrm{MIC}_l + A_l} }^\text {Antibacterial death} \end{aligned} \end{aligned}$$

14 $$\begin{aligned}&\begin{aligned} \frac{\mathrm{d}R_s}{\mathrm{d}t}&= r(1-\alpha _s)\left( 1-\frac{N}{\mu }\right) E_RR_s + R_s(\beta _sS - \beta _lR_l - \beta _uR_u) \\&\quad + \varLambda '(t)\rho _s \frac{\nu }{V(t)} - \frac{\varLambda '(t) R_s}{V(t)} - \\&\quad \delta _RR_s- \delta _lR_s\frac{A_l}{\mathrm{MIC}_l + A_l} \end{aligned} \end{aligned}$$

15 $$\begin{aligned} \frac{\mathrm{d}R_l}{\mathrm{d}t}&= r(1-\alpha _l)\left( 1-\frac{N}{\mu }\right) E_SR_l + R_l(-\beta _sR_s + \beta _lS - \beta _uR_u) \nonumber \\&\quad + \varLambda '(t)\rho _l \frac{\nu }{V(t)} - \frac{\varLambda '(t) R_l}{V(t)} - \delta _RR_l \end{aligned}$$

and

16 $$\begin{aligned} \frac{\mathrm{d}R_u}{\mathrm{d}t}&= r(1-\alpha _u)\left( 1-\frac{N}{\mu }\right) E_RR_u +R_sR_l(\beta _s+ \beta _l) \nonumber \\&\quad + \beta _uR_u(S + R_s + R_l) + \varLambda '(t)\rho _u \frac{\nu }{V(t)} - \frac{\varLambda '(t) R_u}{V(t)} - \delta _RR_u. \end{aligned}$$

Here *N*, *V*(*t*), $E_S$, $E_R$, $\rho$ and $\varLambda$ are given by

17 $$\begin{aligned} N&= S+R_s + R_l + R_u \end{aligned}$$

18 $$\begin{aligned} V(t)&= V_1+\varLambda (t) -\omega (t) \end{aligned}$$

19 $$\begin{aligned} E_S&=1- \frac{E_{\max }A_s^H}{\mathrm{MIC}_s^H+A_s^H} \end{aligned}$$

20 $$\begin{aligned} E_R&= 1- \frac{E_{\max }A_s^H}{\mathrm{MIC}_R^H+A_s^H} \end{aligned}$$

21 $$\begin{aligned} \rho&=\rho _s+\rho _l+\rho _u \end{aligned}$$

22 $$\begin{aligned} \varLambda (t)&= \lambda t. \end{aligned}$$

The initial conditions for bacteriolytic and bacteriostatic antibiotic are $A_{s}{(0)}=A_{l}{(0)}=0$, respectively. Similarly, the initial conditions for the four strains of bacteria are $S(0) = R_{s}(0) = R_{l}(0)=R_{u}(0)=0$. The ODE45 MATLAB solver was used to solve the equations in MATLAB_R2019a.

## Results and Discussion

**Fig. 1:**
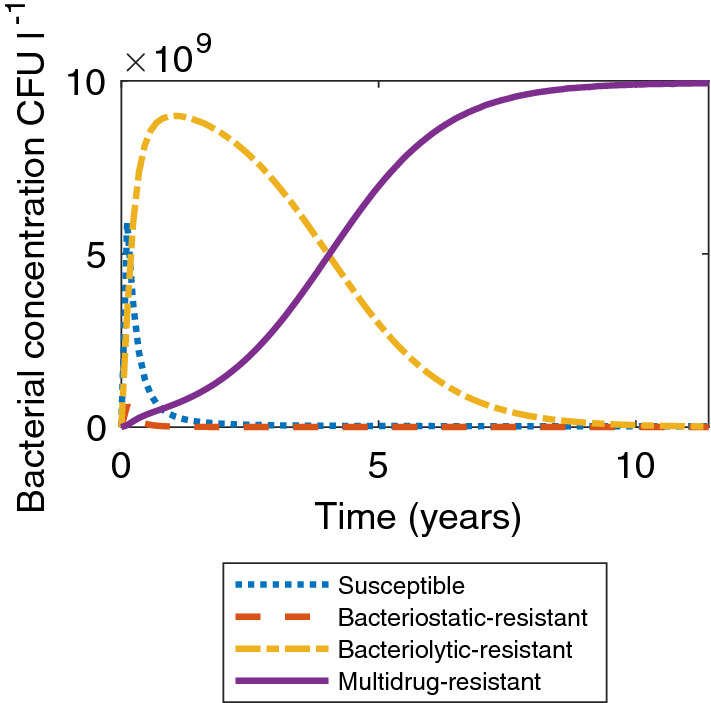
The model simulated using standard parameter values, as in Table 1

Figure 1 shows a simulation of the model under standard parameter values as in Table 1. After ten years, the proportion of multidrug-resistant bacteria is approximately one and the number of bacteria in the tank has reached the carrying capacity. After approximately one year, the tank is dominated by bacteriolytic-resistant bacteria and the community of susceptible bacteria makes up around 5% of the total bacteria in the tank. This is similar to the timescales in Baker et al. (2016), where susceptible bacteria’s presence in the tank is negligible after 300 days. For completeness it should be noted that the apparent steady state shown in Fig. 1 is unstable, but degrades slowly. The stable steady state (susceptible bacteria dominating the tank) is not reached until after 200 years. Furthermore, the addition of a regular emptying regime stabilises the multidrug-resistant dominant state.

**Fig. 2:**
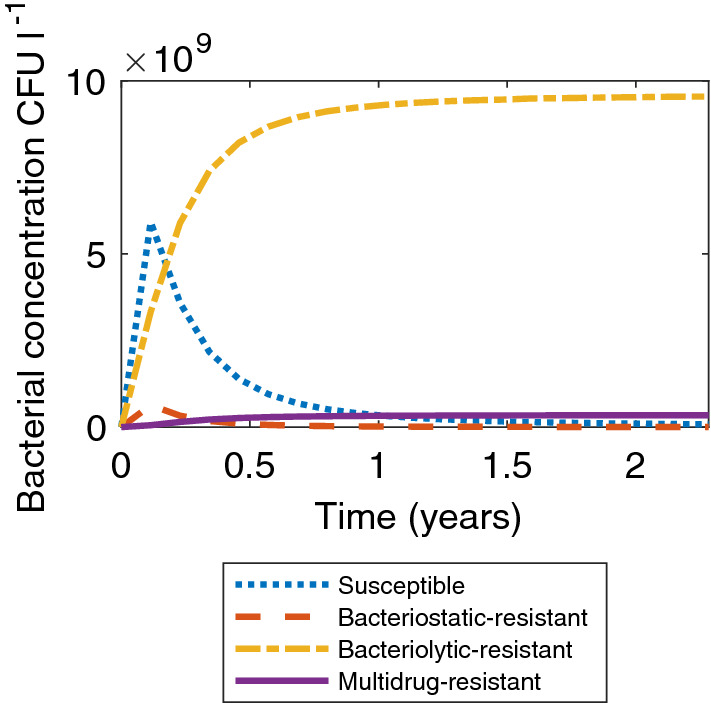
Plot of bacterial concentration when the rate of MDR transfer, $\beta _u$, is zero

**Fig. 3:**
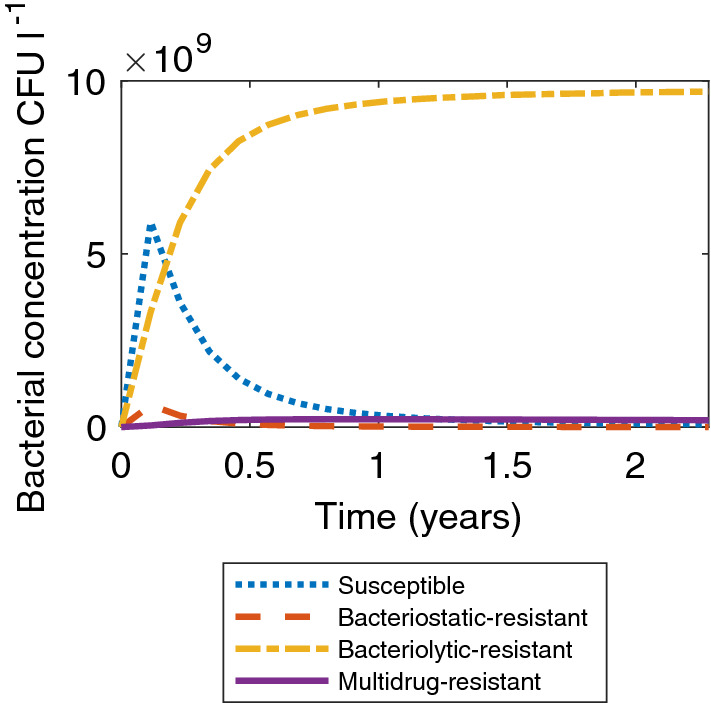
Plot of bacterial concentration when the relative cost of MDR, $\alpha _u$, is 0.2

Certain scenarios using alternative parameter values have also been considered to ensure the model behaves as expected. Figure 2 shows the bacterial concentrations when the rate of multidrug-resistant gene transfer, $\beta _u$, is equal to zero and the other parameters are as in Table 1. Here we assume there is no mobile genetic element that can directly transfer MDR to a bacterium, instead MDR can only be acquired through the acquisition of the two separate genetic elements which confer bacteriostatic and bacteriolytic resistance. This results in bacteriolytic-resistant bacteria dominating the tank, while less than twenty percent of the bacterial population are multidrug-resistant. In this case a small proportion of the tank acquires MDR, while bacteriolytic bacteria make up the greatest proportion in the tank. This simulation acts as a sense-check for the model, as we would expect to see that MDR is less prevalent under these conditions. However, it may not be particularly true to life. As we know that ARGs accumulate on plasmids (Bennett 2008; Van Hoek et al. 2011) and that in the presence of both types of antibiotic there is a biological advantage to acquiring both resistance genes, it is very unlikely that $\beta _u$ would be zero.

Figure 3 shows the bacterial concentrations in the tank when the physiological cost of maintaining MDR, $\alpha _u$, is increased from 0.1 to 0.2 to simulate stacking resistance costs (i.e. $\alpha _u=\alpha _l+\alpha _s$). We have assumed up until this point that the physiological cost of being resistant to a single antibiotic is the same as the physiological cost of being resistant to both from a microbial growth perspective, as if MDR was accompanied with a compensatory mutation which mitigated the cost of being resistant to both antibiotics (Handel et al. 2006; Melnyk et al. 2015). It is also likely that there would be selective pressure to keep the cost of resistance low. In this new scenario we assume there is no compensatory mutation, so that being resistant to both strains of antibiotic causes the relative fitness cost to the microbial population to double. In this scenario, multidrug-resistant bacteria never become the dominant microbial strain the tank and we see similar dynamics to those in Fig. 2 as bacteriolytic-resistant bacteria become the dominant strain within the tank.

### Effects of Altering the Tank Emptying Regime

An aspect that differentiates the current model from previous ones is the inclusion of a tank emptying. Thus far, the volume function, $V(t)=V_1+\lambda t$, has been used (setting the emptying term $\omega (t)=0$), following Baker et al. (2016), in effect simulating an infinite capacity slurry tank. Instead consider a regular, instantaneous emptying regime, whereby the tank fills for a set number of hours, $\tau _e$, then is emptied to its original volume (and not cleaned). If the tank is emptied completely and cleaned, this is the equivalent of running the model up to time $\tau _e$ and then restarting it completely. In this case it is possible to specify an acceptable resistant proportion within the tank and choose $\tau _e$ such that the concentration of resistant *E. coli* never exceeds that proportion. However, this is typically an unrealistic stewardship solution as tank cleaning is costly, not advantageous to the role of the tank and can be a health and safety risk. It also disregards the possible influence of resistant biofilms which could be employing multiple resistance mechanisms (Mah and O’Toole 2001). With this in mind we model emptying without cleaning and for simplicity assume that emptying is instantaneous, so that

23 $$\begin{aligned} \omega (t) = \tau _e\mathrm {floor}\left( \frac{t}{\tau _e}\right) \end{aligned}$$

where $\tau _e$ is the time between tank emptyings, i.e.

24 $$\begin{aligned} V(t) = V_1 + \lambda t -\omega (t) = V_1 + \lambda (t \text { mod }(\tau _e)) \end{aligned}$$

where mod is the modulo function, which finds the remainder when dividing one number by another. A range of $V_1$ and $\tau _e$ values were chosen to simulate the conditions in a farm slurry tank which has a capacity of three million litres. We simulated the conditions for varying $V_1$ and $\tau _e$ over 20 years, the minimum recommended lifespan of a slurry store (Department for Environment, Food and Rural Affairs 2018). Changing the emptying regime in this way leads to the final proportion of multidrug-resistant bacteria varying between 0.976 and 0.992, so that multidrug-resistant bacteria dominate the tank regardless of emptying regime.

**Fig. 4:**
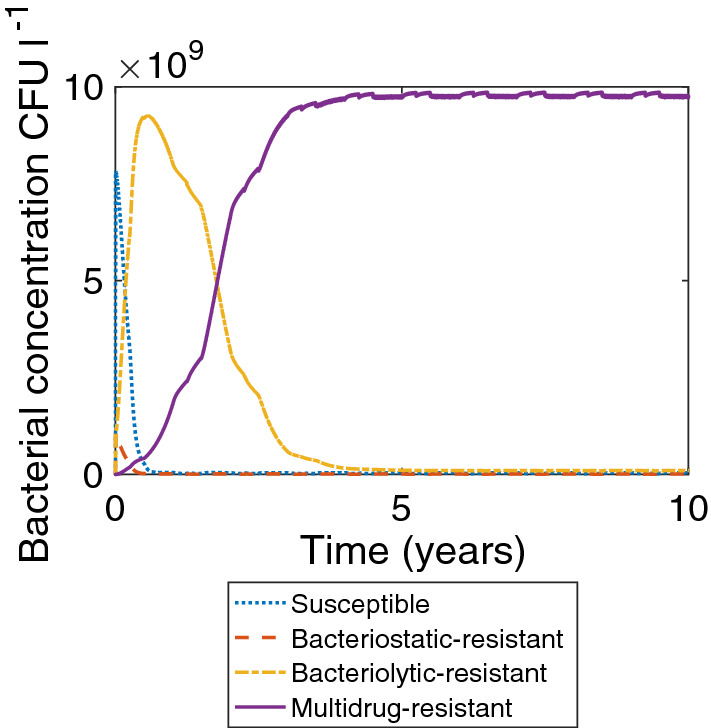
Plot of bacterial concentration when there is a seasonal emptying regime

**Fig. 5:**
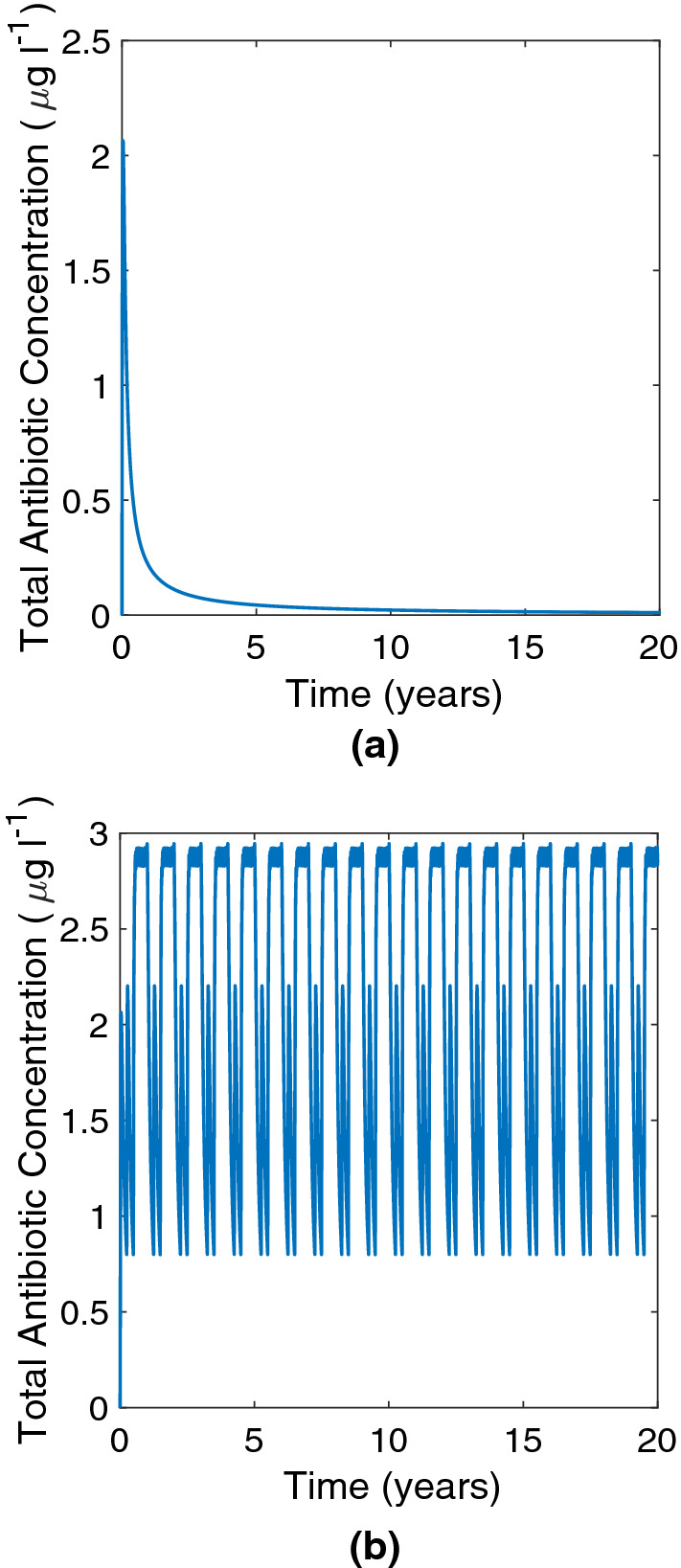
Plots of the total antibiotic concentration in slurry ($A_l + A_s$) under no-emptying and seasonal emptying regimes. **a** Total antibiotic concentration under a no-emptying regime. **b** Total antibiotic concentration under a seasonal emptying regime

However, implementing a seasonal emptying regime based on government guidance for NVZs (every week 1st May to 31st October and every 90 days in the other months, accounting both for the muck spreading closed period between October and February and for those times when the land is more likely to be flooded or frozen (European Community 2001; Department for Environment, Food and Rural Affairs 2018; Department of Agriculture, Environment and Rural Affairs 2019)) leads to multidrug-resistant bacteria dominating the tank within 5 years, as shown in Fig. 4. In the no-emptying case, it took 10 years for multidrug-resistant bacteria to dominate the tank. Figure 5a shows the antibiotic concentration in the no-emptying case, and Fig. 5b shows the antibiotic concentration in the seasonal emptying case. By emptying the tank regularly, a higher concentration of antibiotic is maintained, driving up resistance through environmental pressure. This indicates that implementing an emptying regime has an effect on the speed at which MDR develops, despite having little effect on the eventual proportion of multidrug-resistant bacteria in the tank.

**Fig. 6:**
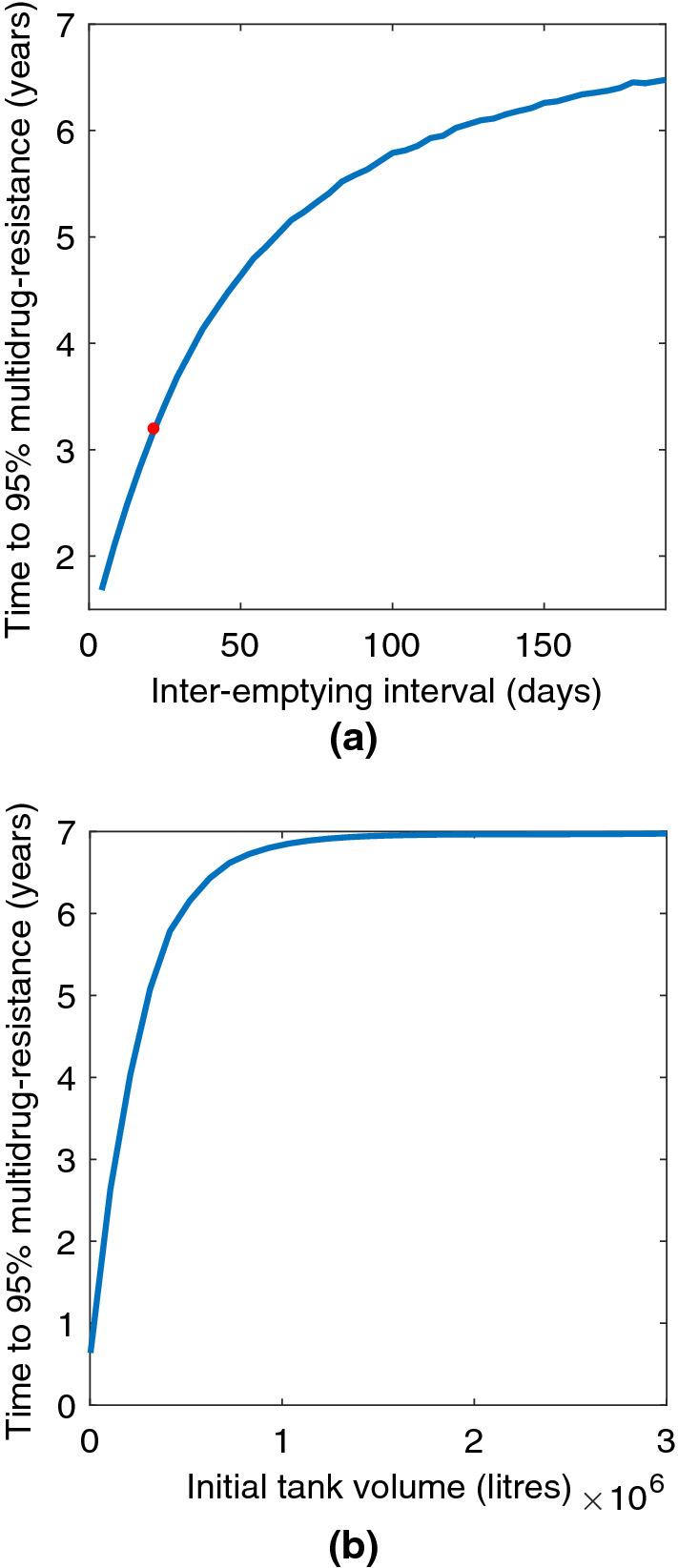
Effects of varying the time between tank emptying, $\tau _e$, and the initial tank volume, $V_1$, on the time to reach 95% multidrug-resistant bacteria within the tank. The dot in 6a indicates the time to 95% MDR using the seasonal emptying frequency. A seasonal emptying regime is being used in 6b. **a** Varying the time between tank emptying, $\tau _e$. **b** Varying the initial tank volume, $V_1$

By plotting the time at which multidrug-resistant bacteria make up 95% of the total population it was possible to visualise the individual effects of the initial volume $V_1$ and of the time to empty, $\tau _e$. Figure 6 shows that as $\tau _e$ and $V_1$ increase so does the time to reach 95% MDR. This indicates that farm practice can have an influence on the way in which AMR persists and spreads within the slurry tank. The time at which multidrug-resistant bacteria make up 95% of the population under an NVZ seasonal emptying regime is marked in Fig. 6a. Figure 6b shows the relationship between the initial tank volume and the time to 95% MDR. The higher the initial volume, the longer it takes for multidrug-resistant bacteria to dominate the tank.

**Fig. 7:**
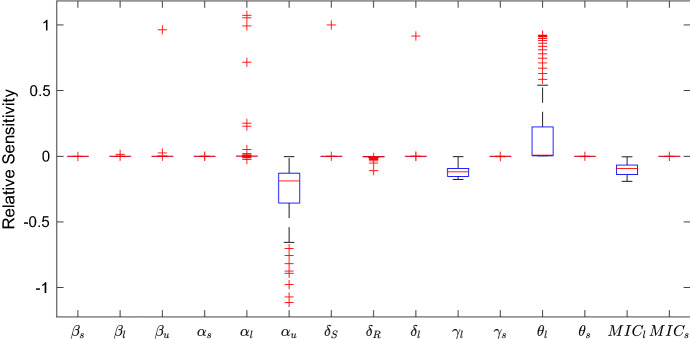
Sensitivity analysis of the model parameters where the proportion of multidrug-resistant bacteria is the output of interest under the no emptying regime. See Table 1 for parameter definitions. The system is particularly sensitive to $\alpha _u$, the relative cost of multidrug resistance and $\theta _l$ the inflow rate of bacteriolytic antibiotic. It is also sensitive to other parameters relating to bacteriolytic antibiotics

Current legislation dictates that slurry can only be spread between 1st February and 31st October, and only during times at which the ground is not frozen or at risk of flooding during the other months (European Community 2001) meaning that the slurry store is emptied more frequently during the summer months. According to our simulations, if the tank is emptied to 150,000 litres and a seasonal emptying regime is used, then it takes just over three years for multidrug-resistant bacteria to make up 95% of the population in the tank, as shown in Fig. 6a. Alternatively, if the minimum volume of slurry in the tank is maintained at around 1.5 million litres, the time to MDR dominance could be increased to seven years without having to alter the rate at which the tank is emptied, see Fig. 6b.

### Sensitivity Analysis

In order to provide a more precise analysis of how model parameters affect the output of the model, one-at-a-time sensitivity analysis has been conducted. This is a process that allows the investigation of the sensitivity of a model’s output to its individual input parameters (Pianosi et al. 2016). Sensitivity scores, $S_X$ where *X* is the parameter being changed, were calculated for fifteen model parameters as shown in Table 2; the model output being measured was the proportion of multidrug-resistant bacteria in the tank. Here the sensitivity measure $S_X$ is defined by

25 $$\begin{aligned} S_X = \frac{\varDelta P_{SR}/P_{SR}}{\varDelta X/X} \end{aligned}$$

where $P_{SR}$ is the proportion of multidrug-resistant bacteria in the tank at the end of a standard parameter value simulation, *X* is the parameter being changed and $\varDelta X$ and $\varDelta P_{SR}$ represent the change in the parameter value and the resulting change in the model output, respectively (Saltelli et al. 2008; Baker et al. 2016). $S_X$ was calculated for each parameter at 100 points evenly spaced across the parameter space. Two regimes were considered for sensitivity analysis: a simulation with no tank emptying and a simulation with tank emptying every week between 1st May and 31st October and every 90 days in the other months. Both simulations ran for an in-model time of 20 years.

**Table 2:** The parameters considered for sensitivity testing and the ranges used

Parameter	Range	References
$\beta _s$	0–10$^{-12}\ \mathrm {l CFU}^{-1}\mathrm {h}^{-1}$	Baker et al. (2016), Zhong et al. (2010)
$\beta _l$	0–10$^{-12} \ \mathrm {l CFU}^{-1}\mathrm {h}^{-1}$	Baker et al. (2016), Zhong et al. (2010)
$\beta _u$	0–10$^{-12} \ \mathrm {l CFU}^{-1}\mathrm {h}^{-1}$	Baker et al. (2016), Zhong et al. (2010)
$\alpha _s$	0–1	Full mathematically plausible range
$\alpha _l$	0–1	Full mathematically plausible range
$\alpha _u$	0–1	Full mathematically plausible range
$\delta _S$	0–1 $\hbox {h}^{-1}$	Ayscue et al. (2009)
$\delta _R$	0–1 $\hbox {h}^{-1}$	Ayscue et al. (2009)
$\delta _l$	0–3 $\hbox {h}^{-1}$	Norcia et al. (1999)
$\gamma _l$	0–0.029 $\hbox {h}^{-1}$	Dolliver et al. (2008)
$\gamma _s$	0–0.029 $\hbox {h}^{-1}$	Dolliver et al. (2008)
$\theta _l$	0–3422 $\mu$g $\hbox {h}^{-1}$	Baker et al. (2016)
$\theta _s$	0–3422 $\mu$g $\hbox {h}^{-1}$	Baker et al. (2016)
MIC$_l$	0–250 $\mu$g $\hbox {l}^{-1}$	Bengtsson-Palme and Larsson (2016)
MIC$_s$	0–250 $\mu$g $\hbox {l}^{-1}$	Bengtsson-Palme and Larsson (2016)

Figure 7 shows boxplots of the sensitivities for all thirteen parameters in the no emptying regime.

**Fig. 8:**
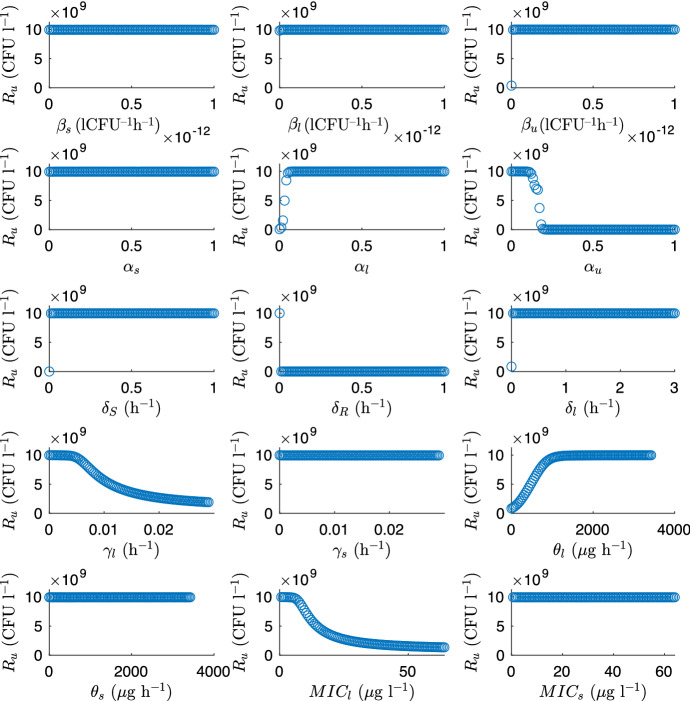
The quantity of multidrug-resistant bacteria, $R_u$, after 20 years as each of the parameters in Table 2 varies

In this model the most sensitive parameters are the relative cost of MDR ($\alpha _u$), the relative cost of bacteriolytic resistance ($\alpha _l$) and the inflow rate of bacteriolytic antibiotic ($\theta _l$). The model is also sensitive to the bacteriolytic MIC (MIC$_l$) and the decay rate of bacteriolytic antibiotic ($\gamma _l$), indicating that it is the parameters relating to bacteriolytic antibiotics that most affect the development of multidrug-resistant bacteria. Figure 8 shows how changing each parameter affects the proportion of multidrug-resistant bacteria. It is clear that the most sensitive parameters have the largest affect on the proportion of $R_u$ bacteria. Increasing the costs of bacteriolytic resistance or MDR ($\alpha _l$ and $\alpha _u$, respectively) decreases the total multidrug-resistant community. In the case of $\alpha _u$ this occurs very drastically at around $\alpha _u$=0.2, indicating that within the model this is a maximum tolerable cost of MDR. Similarly increasing the death rate of resistant bacteria ($\delta _R$) causes a complete removal of multidrug-resistant bacteria. Increasing the inflow of bacteriolytic antibiotic ($\theta _l$) increases the population of multidrug-resistant bacteria, due to increased selective pressure, whereas increasing the MIC of bacteriolytic antibiotic (MIC$_l$) decreases the number of multidrug-resistant bacteria. The MIC increases the effect of the antibiotic on the susceptible bacteria decreases, allowing them to live for longer in the tank.

**Fig. 9:**
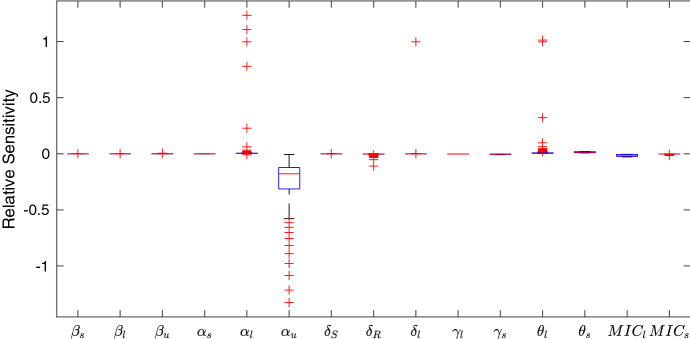
Sensitivity analysis of the model parameters where the proportion of multidrug-resistant bacteria is the output of interest and a seasonal emptying regime is used. See Table 1 for parameter definitions. Compared to the infinite tank simulations the system is much less sensitive (see Fig. 7), but $\alpha _u$, the relative cost of multidrug resistance remains the most sensitive parameter. Similarly, the system is also sensitive to $\theta _l$, the inflow rate of bacteriolytic antibiotic, and $\alpha _l$, the cost of bacteriolytic resistance

**Fig. 10:**
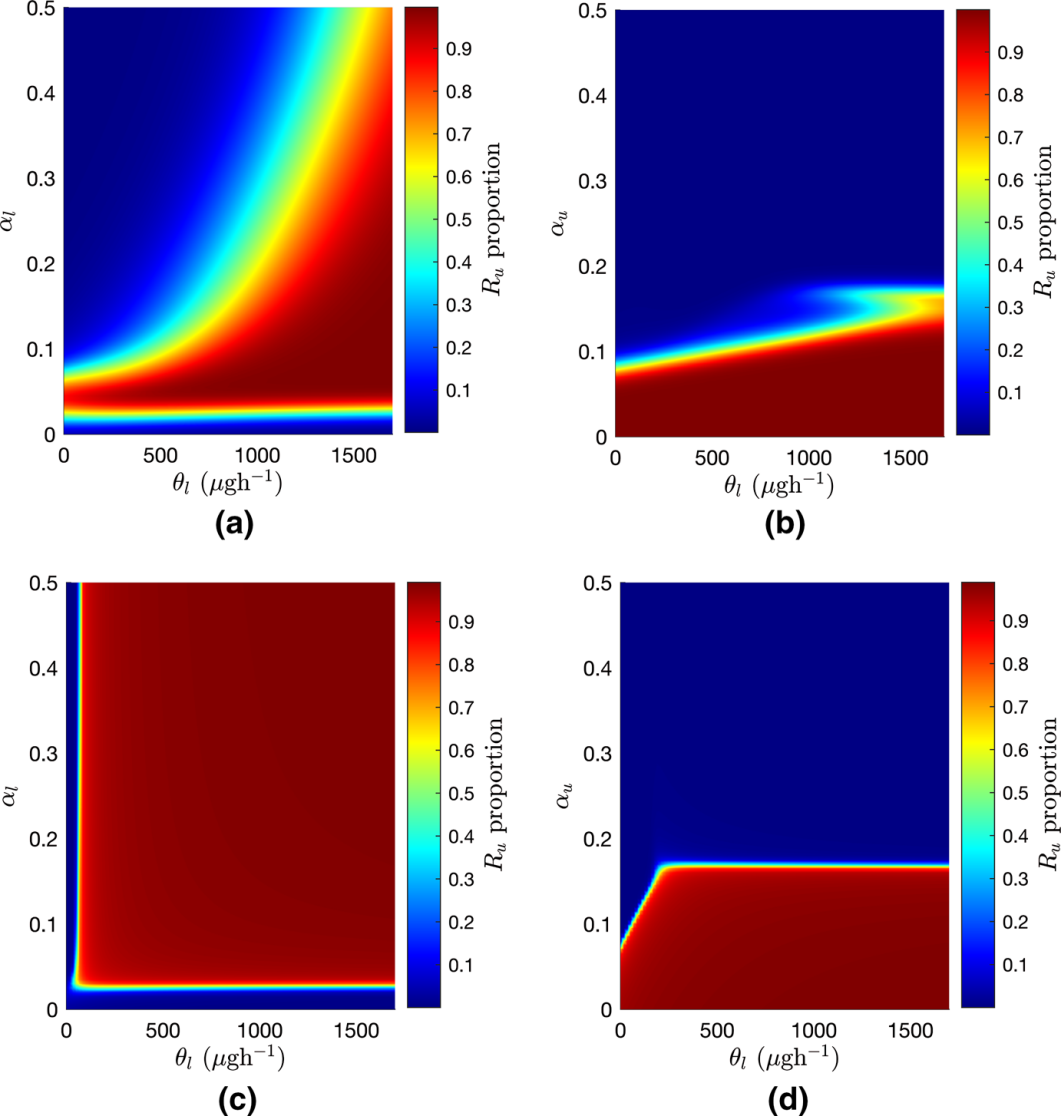
Heatmaps showing the multidrug-resistant proportion in the tank after 20 years when varying the relative cost of bacteriolytic resistance, $\alpha _l$, and the relative cost of MDR, $\alpha _u$, against the inflow rate of bacteriolytic antibiotic, $\theta _l$, under no-emptying and seasonal emptying regimes. **a** Relative cost of bacteriolytic resistance, $\alpha _l$, plotted against the inflow rate of bacteriolytic antibiotic, $\theta _l$, under a no-emptying regime. **b** Relative cost of MDR, $\alpha _u$, plotted against the inflow rate of bacteriolytic antibiotic, $\theta _l$, under a no-emptying regime. **c** Relative cost of bacteriolytic resistance, $\alpha _l$, plotted against the inflow rate of bacteriolytic antibiotic, $\theta _l$, under a seasonal emptying regime. **d** Relative cost of MDR, $\alpha _u$, plotted against the inflow rate of bacteriolytic antibiotic, $\theta _l$, under a seasonal emptying regime

**Fig. 11:**
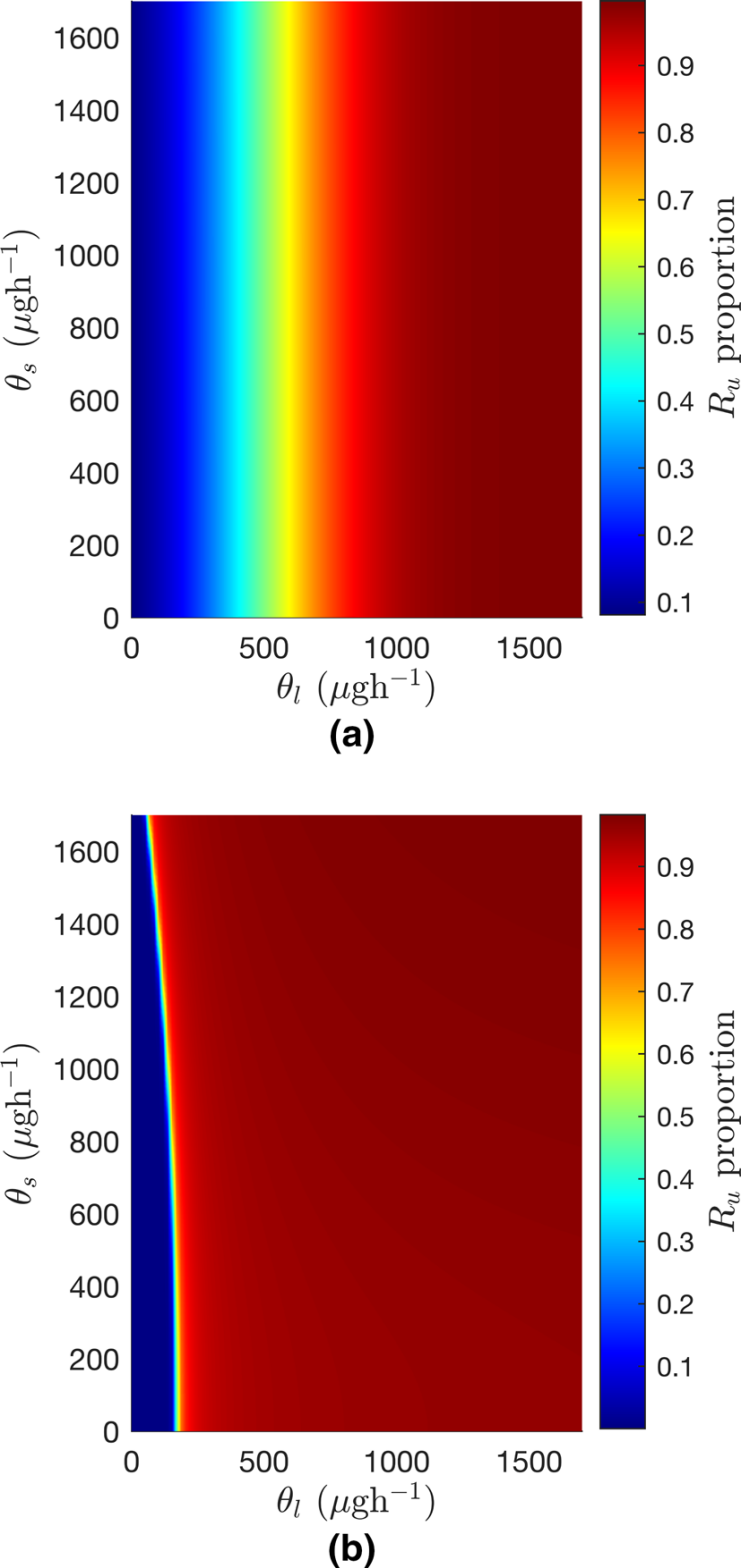
Heatmaps showing the multidrug-resistant proportion in the tank after 20 years when varying the inflow rate of bacteriostatic antibiotic, $\theta _s$, and the inflow rate of bacteriolytic antibiotic, $\theta _l$, under a no-emptying regime and a seasonal emptying regime. **a** Inflow rate of bacteriostatic antibiotic, $\theta _s$, plotted against the inflow rate of bacteriolytic antibiotic, $\theta _l$, under a no-emptying regime. **b** Inflow rate of bacteriostatic antibiotic, $\theta _s$, plotted against the inflow rate of bacteriolytic antibiotic, $\theta _l$, under a seasonal emptying regime

Figure 9 shows the relative sensitivities of the fifteen parameters under a seasonal emptying regime (every week 1st May to 31st October and every 90 days in the other months). In this scenario the system’s sensitivity to $\alpha _l$, $\theta _l$, MIC$_l$ and $\gamma _l$ decreases, while the system remains similarly sensitive to the natural death rate of resistant bacteria, $\delta _R$.

Sensitivity analysis of this model indicates that the mechanisms that most affect the proportion of multidrug-resistant bacteria in the slurry tank are the concentration of bacteriolytic antibiotic and the relative cost of resistance to bacteria. This indicates that the ramifications of the use of bacteriolytic antibiotics in farming, such as penicillins and cephalosporins, should be investigated.

### Bacteriolytic and Bacteriostatic Antibiotics

Sensitivity analysis indicates that some of the most sensitive parameters in the system are those related to the properties of bacteriolytic antibiotic. As one-at-a-time sensitivity analysis shows the effects of changes to one parameter, we have used heatmaps to show the effect of altering the bacteriolytic antibiotic parameters in tandem with the cost of bacteriolytic and multidrug resistance under a no-emptying regime, and with a seasonal emptying regime, shown in Fig. 10.

From Fig. 10a we can see the effects of varying $\alpha _l$ and $\theta _l$ simultaneously under an no-emptying regime. For $\alpha _l<$0.05 the growth of bacteriolytic-resistant bacteria is uninhibited and they dominate the tank, meaning that $P_{SR}\approx 0$. As $\alpha _l$ increases, higher quantities of antibiotic need to be introduced into the system to apply enough environmental pressure to select for multidrug-resistant bacteria. Varying $\alpha _u$ alongside $\theta _l$ under a no emptying regime shows that, as $\theta _l$ increases, the bacterial tolerance for the cost of MDR increases until once $\alpha _u$ reaches a threshold value of approximately 0.2; see Fig. 10b. When $\alpha _u>0.2$ $P_{SR}\approx 0$ for all values of $\theta _l$, with the increased relative cost for the growth of multidrug-resistant bacteria counteracting the selective pressure of the antibiotic.

When we vary $\alpha _l$ and $\theta _l$ simultaneously under an seasonal emptying regime, as shown in Fig. 10c, we see that, even for rates of bacteriolytic inflow of less than $100\mu \hbox {g}$ $\hbox {h}^{-1}$ the MDR can develop even when the relative cost of bacteriolytic resistance is 0.5. In the no-emptying case, for the same inflow rate of bacteriolytic antibiotic, the development of MDR required the cost of bacteriolytic resistance to be around 0.05; for $\alpha _l>0.1$ multidrug-resistant bacteria did not dominate the tank. This change is likely because under the seasonal emptying regime the long-term concentration of antibiotic is increased and there is more selective pressure on the bacteria in the tank. Figure 10d shows the $R_u$ proportion as $\alpha _u$ and $\theta _l$ vary. The bacterial tolerance for the cost of MDR increases as $\theta _l$ increases up to around $\theta _l=250 \mu$g $\hbox {h}^{-1}$, and then reaches a threshold value of around $\alpha _u=.18$.

Figure 11 shows the $R_u$ proportion after 20 years for varying $\theta _l$ and $\theta _s$ under no-emptying and seasonal emptying regimes. From this figure it is very clear that the inflow rate of bacteriostatic antibiotic has little effect on the eventual proportion of multidrug-resistant bacteria. On the other hand, increasing the inflow rate of $A_l$ has a marked influence on the $R_u$ proportion. This is not to say, however, that $\theta _s$ has no effect on the system: as $\theta _s$ decreases the time it takes for $R_u$ to dominate the tank increases.

### Discussion and Recommendations for Future Work

This model of AMR in the dairy slurry tank predicts the emergence of widespread MDR after a decade of use. It also predicts that changes in agricultural practice could delay the rate at which MDR develops and shows that farm practice is an important consideration for those modelling AMR in agricultural settings.

We have shown that although implementing a tank emptying regime into a model does not affect the proportion of multidrug-resistant bacteria in the long term, it does change the speed at which model equilibrium is reached. This is interesting from a farm practice perspective as it implies that it is safer to store slurry in higher volumes for longer periods of time and when it is removed, only to remove it in small quantities.

To better understand the model behaviour, a sensitivity analysis was performed to determine which parameters most affect the model output. Two scenarios were considered: a simulation with no tank emptying regime, and one with seasonal tank emptying. In both cases the system is most sensitive to the relative costs of multidrug and bacteriolytic resistance $\alpha _u$ and $\alpha _l$, and to the inflow rate of bacteriolytic antibiotic, $\theta _l$. This brings about another possible implication for farm practice, indicating that reduced usage of bacteriolytic antibiotics would decrease the number of multidrug-resistant bacteria much more effectively than reducing the quantity of bacteriostatic antibiotics used on farms. Thus, the model appears to advise two possible channels for research into the way that farm practices affect the persistence and spread of AMR within the slurry tank; the storage and emptying regime and the administration of bacteriolytic antibiotics such as beta-lactam antibiotics, for example penicillin, or cephalosporins to the herd.

When considering the cost of resistance, we have generally assumed that the cost of MDR, $\alpha _u$, is the same as the cost of resistance to a single type of antibiotic, either due to a compensatory mutation (Handel et al. 2006; Melnyk et al. 2015) or the fact that the cost of resistance is likely to be under selective pressure; a version of the resistance gene which incurs a low fitness cost will outcompete those with higher fitness costs.

It is important that the effects of slurry storage on the spread of AMR are further investigated. Our model has shown that both the volume of slurry left in the tank and the frequency at which the tank it is emptied affect the rate at which MDR develops. We recommend a longitudinal survey and analysis of AMR in slurry at the point of land application after various storage times. Furthermore, slurry stores vary in design, from clay-lined lagoons to steel towers or concrete stores (AHDB Dairy 2019); we currently have little knowledge on how the storage design might affect the development of AMR. In our analysis we have assumed that the slurry tank is never cleaned or disinfected. If it is, this could delay the process of multidrug-resistant development, as, from a modelling perspective, it would be the same as restarting the model at day zero. However, if resistant biofilms (Mah and O’Toole 2001) develop in the tank and are not fully cleaned away, they could potentially act as a seed for resistance in the tank, as biofilms are known to promote HGT of ARGs (Savage et al. 2013; Balcázar et al. 2015). Another farm practice factor which requires more investigation is the effect of heavy metal co-resistance on bacterial populations in slurry, given the widespread use of copper and zinc footbaths in the UK (Pal et al. 2017; Williams et al. 2019). Though it is well known that metals can contribute to the development and spread of AMR, it has also been shown that heavy metals can impede selection for resistance, in the case of ciprofloxacin and zinc (Vos et al. 2019). A future model could consider cases in which heavy metals both contribute and detract from the development of AMR.

There are various environmental factors that have been shown to have an impact on the development of AMR that were omitted in the model. The temperature and pH of the slurry will likely affect the growth of microbes other than *E. coli* (Kearney et al. 1993; Biswas et al. 2016) and there is evidence that there is seasonal variation in AMR spread in swine agriculture (Chen et al. Jun 2010; Awad et al. 2013; Sui et al. 2015). To model other microbial strains, temperature dependence would likely need to be added to the natural death terms $\delta _R$ and $\delta _S$. We have assumed that the tank is uncovered, but that slurry dilution by rainfall is negligible. However, there is potentially significant dilution to the slurry via rainfall which may affect the rate at which resistance develops. Though our model incorporates farm practice elements we have not considered these environmental factors, which may play a crucial role in agricultural AMR.

Though we have assumed in this model that the slurry is well mixed, given that slurry stores are often large (potentially around three-million litres (AHDB Dairy 2019)), the tanks are not continuously stirred, and the input to the tank is changeable, this is unlikely to be the case. Thus, we recommend an extension of this model to take into account spatial factors, including the position of the mixing mechanism and the inflow and outflow pipes to the tank.

We have consolidated all factors affecting HGT into a scalar rate coefficient; however, there is evidence that HGT occurs with a degree of stochasticity (Lawrence and Ochman 2002) and therefore may be better modelled under a stochastic framework. Furthermore, one of the mechanisms of HGT is the acquisition of extracellular DNA released by dead bacteria (Thomas and Nielsen 2005), which is not considered in this model.

The development of MDR in the model is particularly sensitive to the inflow rate of bacteriolytic antibiotic (see Figs. 7, 9 and 11); however, as the half-life of bacteriolytic antibiotic and the relative cost of bacteriolytic resistance increase, the multidrug-resistant population in the tank decreases, see Fig. 8. This implies that the chemical properties of the antibiotics being used also have an effect on whether or not MDR develops.

Though there are few studies to which we can accurately compare our model, it generates comparable results to the experimental study conducted by Mulamattathil et al. (2000), which sampled faecal coliform bacteria from a water reticulation system near a chicken meat processing plant. Their findings show population-wide resistance developing over similar timescales to that of the model, alongside stable populations of multidrug-resistant bacteria, similar to the tail end of the simulation shown in Fig. 1. Though the system sampled from is quite different to our model system, in that it is comprised of multiple water storage facilities, the similarities between the results are encouraging. As the study does not sample only *E. coli*, temperature may play some part in the development of resistance at each of the sampling points. Furthermore, the fluctuations in resistance to specific antibiotics shown by Mulamattathil et al. (2000) indicate that an interesting extension to the model would be to incorporate resistance to multiple specific bacteriolytic and bacteriostatic antibiotics with different modes of action. In addition, Gullberg et al. (2011) found that in certain *E. coli* strains, when exposed to varying concentrations of ciprofloxacin, resistant bacteria could outnumber susceptible by over 100 to 1 after 60 generations of growth over 24 h, though the speed of growth was found to vary with strain and antibiotic concentration. This indicates that it is plausible that resistant bacteria can dominate systems in which selective pressure is exerted by antibiotics. To test the outputs of our model in more detail, time series data from dairy slurry storage facilities will need to be analysed; such data are not yet available.

## Conclusions

We have developed a model of the spread and persistence of antimicrobial resistance in an *E. coli* population in a dairy farm slurry tank. The model incorporates bacteriolytic and bacteriostatic antibiotics and four strains of *E. coli*: a strain susceptible to both antibiotics, a strain resistant to bacteriolytic antibiotics, a strain resistant to bacteriostatic antibiotics and a “multidrug-resistant” strain which is resistant to both antibiotics. We have shown that tank emptying regime may affect the rate at which AMR develops. We have also shown that bacteriolytic antibiotics have more effect on the spread of MDR than bacteriostatic antibiotics.
